# Supplementary material for: Dynamic transcriptomic profiles of zebrafish gills in response to zinc supplementation
Source: BMC Genomics. 2010 Oct 11;11:553. doi: 10.1186/1471-2164-11-553 (PMC3091702; doi:10.1186/1471-2164-11-553)
Supplement: Additional file 2 — Interactive Direct Interaction Network representing the molecular interactions between zinc, copper, iron, calcium and proteins encoded by transcripts changed by zinc supplementation. Mini web-site containing index.html and hyperlinked pages in subdirectory describing a Direct Interaction Network automatically generated based on curated interactions contained within the proprietary PathwayArchitect database. Ovals represent proteins and the circles symbolize metal ions. Objects are coloured by their abundance in zebrafish at the time-point they were significantly different from the control is a scale from -4 fold (dark green) to +4 fold (dark red). Where significant differences were found at more than one time-point, the colour overlay shows expression at the first instance. Dark blue squares denote 'binding', and light blue squares 'expression'; green squares stand for 'regulation', green diamonds for 'metabolism', and green circles for 'promoter binding'. Arrow heads indicate directionality of the interaction where annotated. All nodes and edges can be further interrogated by selecting the relative area of the image. [file 1471-2164-11-553-S2.zip › PathwayArchitect Zn xs DIN/1474866.html]

# REGULATION:

|  |  |
| --- | --- |
| Type | REGULATION |
| Effect | None |


---

|  |  |
| --- | --- |
| Score | 0 |


---

|  |  |
| --- | --- |
| Reference Count | 5 |


---

|  |  |
| --- | --- |
| Mechanism | Unknown |


---

|  |  |
| --- | --- |
| Reference:0 || Sentence | "These data indicate that tissue copper deficiency does not alter FPN1 expression but that copper adequacy may be required for appropriate regulation of FPN1 by iron status." |
| PMID | 14988440 |
| Year | 2004 |
| Species | Mouse |
| Journal | J Nutr |
| RefScore | 1 |
| Source | PArchNLP |
  |
|  |  |


---

|  |  |
| --- | --- |
 Reference:1 || Sentence | TNFalpha mediated an early induction in both iron import and iron export, which were associated with increased DMT-1 and IREG-1 mRNA and protein expression (P<0.05). |
| Year | 2005 |
| PMID | 15901240 |
| Species | Human |
| Journal | Biochem J |
| RefScore | 1 |
| Source | PArchNLP |
  ||  |  |


---

|  |  |
| --- | --- |
 Reference:2 || Sentence | This report describes the clinical and laboratory findings of two Spanish families with autosomal dominant iron overload associated with previously unrecognized Ferroportin 1 mutations (p.R88T and p.I180T). |
| Year | 2006 |
| PMID | 16257244 |
| Species | Human |
| Journal | Blood Cells Mol Dis |
| RefScore | 0 |
| Source | PArchNLP |
  ||  |  |


---

|  |  |
| --- | --- |
 Reference:3 || Sentence | Autosomal dominant iron overload due to a novel mutation of ferroportin1 associated with parenchymal iron loading and cirrhosis. |
| Year | 2004 |
| PMID | 15030991 |
| Species | Human |
| Journal | J Hepatol |
| RefScore | 1 |
| Source | PArchNLP |
  ||  |  |


---

|  |  |
| --- | --- |
 Reference:4 || Sentence | Dcytb, DMT1, Ireg1 and transferrin receptor 1 mRNA expression in the spleen and liver of mice treated with PHZ responded to the enhanced iron demand associated with the resulting stimulation of erythropoiesis. |
| PMID | 15469906 |
| Year | 2004 |
| Species | Mouse |
| Journal | Biochim Biophys Acta |
| RefScore | 2 |
| Source | PArchNLP |
  |


---

|  |  |
| --- | --- |
